# Anticorrosion Behaviour of* Rhizophora mangle* L. Bark-Extract on Concrete Steel-Rebar in Saline/Marine Simulating-Environment

**DOI:** 10.1155/2019/6894714

**Published:** 2019-08-19

**Authors:** Joshua Olusegun Okeniyi, Jacob Olumuyiwa Ikotun, Esther Titilayo Akinlabi, Elizabeth Toyin Okeniyi

**Affiliations:** ^1^Mechanical Engineering Department, Covenant University, Ota 112001, Nigeria; ^2^Department of Mechanical Engineering Science, University of Johannesburg, Johannesburg 2006, South Africa; ^3^Department of Civil Engineering and Building, Vaal University of Technology, Vanderbijlpark 1911, South Africa; ^4^Petroleum Engineering Department, Covenant University, Ota 112001, Nigeria

## Abstract

This paper investigates anticorrosion behaviour of the bark-extract from* Rhizophora mangle* L. on steel-rebar in concrete slabs in 3.5% NaCl medium of immersion (for simulating saline/marine environment). Corrosion-rate, corrosion-current, and corrosion-potential were measured from the NaCl-immersed steel-reinforced concrete cast with admixture of different plant-extract concentrations and from positive control concrete immersed in distilled water. Analyses indicate excellent mathematical-correlation between the corrosion-rate, concentration of the bark-extract admixture, and electrochemical noise-resistance (ratio of the corrosion-potential standard deviation to that of corrosion-current). The 0.4667%* Rhizophora mangle* L. bark-extract admixture exhibited optimal corrosion-inhibition performance,* η* = 99.08±0.11% (experimental) or* η* = 97.89±0.24% (correlation), which outperformed the positive control specimens, experimentally. Both experimental and correlated results followed Langmuir adsorption isotherm which suggests prevalent physisorption mechanism by the plant-extract on the reinforcing-steel corrosion-protection. These findings support* Rhizophora mangle* L. bark-extract suitability for corrosion-protection of steel-rebar in concrete structure designed for immersion in the saline/marine environmental medium.

## 1. Introduction

Reinforcing-steel (i.e., steel-rebar) corrosion in concrete is a critical problem militating against structural durability, integrity, and service-life of steel-reinforced concrete building and infrastructure and for which induced maintenance and repair constitute costly parts of budgets in many countries [1–5]. Embedded steel-rebar in concrete corrodes due to chloride ions ingress into concrete from artificial saline (e.g., man-made use of deicing salt in temperate region) or from natural marine from coastal service-environment of the steel-reinforced concrete applications [6, 7]. Active concrete steel-rebar corrosion, due to chloride contamination, produces expansive rusts within the concrete, which can lead to cracks, spalling, as well as delamination deterioration of the steel-reinforced concrete member [4, 8]. The consequent loss of structural integrity at this stage requires costly repair and rehabilitation for it not to culminate in calamitous collapse of the structural steel-reinforced concrete, with serious risks of safety to life and destructions of properties. Averting these and the consideration that steel-reinforced concrete material still remains a choice material, worldwide, for structural and infrastructural building applications, based on its versatility and comparative low cost, necessitate search for effective steel-rebar corrosion-protection methods.

In the past decade, numerous studies have deliberated on corrosion problems that are encountered from the in-service environments for steel-reinforced concrete applications [9–30]. These studies lend supports to the knowledge that the environments for steel-reinforced concrete attacks constitute the acidic [9, 12, 16, 30], alkaline [11, 15, 17, 23] and neutral [14, 18, 29] media, wherein environmental agents such as carbonation [20, 25, 28], chloride ions [10, 19, 21, 24], and sulphate ions [9, 13, 17] effects are indicated for the corrosion degradation mechanisms against steel-in-concrete. These have engendered monitoring techniques [26, 27] and solution approaches [20, 22, 29], over the years, for assessing and/or addressing different modes of reinforcing-steel corrosion in concrete. Some of the proffered solutions include the use of fibre reinforced polymer, stainless steel, protective coating, concrete pore sealers, and corrosion inhibitor as admixture in concrete [14, 26, 31–39].

Among these corrosion mitigation methods, the corrosion inhibitor admixture in concrete is receiving great attention because it is among the most cost-effective and easily applied technique of tackling concrete steel-rebar corrosion in aggressive environments [14, 19, 22, 23, 29, 30, 40, 41]. However, problems persist on corrosion inhibitor usage, especially, due to the fact that well-known substances for inhibiting corrosion of steel-rebar, such as chromates and nitrites, also exhibited toxicity and hazardousness to the environmental ecosystems [41–43]. Consequently, there are restrictions in many countries against their use, thus, necessitating research works for studying effect of novel, natural and eco-friendly admixture substances on corrosion of steel-reinforcement in concrete for aggressive environments [42, 44–46].

Bark-extract of* Rhizophora mangle* L. (*R. mangle* L.),* Rhizophoraceae*, has been identified in studies [47, 48] as exhibiting no toxicity effect to living organisms even as this natural plant extract is also known to have many medicinal benefits for human [49–51]. Studies indicated that* R. mangle* L. bark-extract is rich in tannins [47, 48, 51], from which it could be noted that tannins from* Rhizophora apiculata* (*R. apiculata*), a plant sharing same* Rhizophoraceae* taxonomical family, have been reported as effective inhibitor of steel corrosion [52–54]. Also, recent scientific journals [55, 56] via collaborative work by authors of this study with other researchers, have detailed that* R. mangle* L. leaf-extract and* R. mangle* L. bark-extract are constituted of lone-pairs/*π*-electron rich, organic, biochemical compounds [45]. The constitution of the biochemical compounds detailed in [55, 56] in the leaf and bark of* R. mangle *L. portend corroborations for the positive performances exhibited by the extracts on the inhibition of reinforcing-steel corrosion in sulphuric acid-immersed concrete [57–61]. However, while* R. mangle *L. leaf-extract has also been applied for effectively inhibiting reinforcing-steel corrosion in NaCl-immersed concrete [62, 63], no study have used* R. mangle *L. bark-extract for corrosion-inhibition of steel-rebar in this medium. This is unlike* R. apiculata*, which had been employed, along with phosphoric acid, in the cited work by Rahim et al. [53] for inhibiting corrosion of prerusted steel in 3.5% NaCl. Also, that the steel employed in [53] was not in physically cast concrete necessitates further research needs for assessing anticorrosion performance by this natural plant within hydrated cement pastes, the real-time service-environment of steel-reinforcement in concrete, as recommended from [64–66]. Therefore, the objective of this paper was to investigate the anticorrosion behaviour of* R. mangle* L. bark-extract on steel-rebar embedded in concrete that was physically cast and then immersed in 3.5% NaCl, for the saline/marine steel-reinforced concrete in-service-environment simulation.

## 2. Materials and Methods

### 2.1. Materials

#### 2.1.1. Bark-Extract Admixture from Rhizophora mangle L.

The collection of the barks of* R. mangle* L. at Ehin-more, Ilaje Ese-odo, in Ondo State, Nigeria, its Herbarium identification at the Forestry Research Institute of Nigeria, Ibadan, Oyo State, Nigeria, and the depositing of its voucher FHI No 109501 at the institute was as detailed in [56, 58, 61]. The barks were dried under cover, finely blended into powder [54] and extracted via methanol (CH_3_OH) usage for solvent [67] in a condenser equipped Soxhlet extractor. This was followed by concentrating the obtained methanolic solution over water bath and from which the thick reddish-brown residue finds usage for admixture in the concrete specimens for the study.

#### 2.1.2. Specimens of Concrete Slabs with Steel-Rebar Embedment

Ø12 ribbed steel bar for the study was sourced from Federated Steel Rolling Mill®, Ota, Ogun State, Nigeria. The composition (%) of the rebar includes 0.27 C, 0.40 Si, 0.04 P, 0.78 Mn, 0.04 S, 0.11 Ni, 0.14 Cr, 0.24 Cu, 0.02 Mo, 0.01 Sn, 0.01 Nb, 0.01 Co and the balance Fe. The steel bar was cut lengthwise into 190 mm rods and surface preparations and treatments were maintained, for each of the rods, uniformly, and these were done according to the standard procedures in ASTM G109–07 [68] and which had been described in studies [69, 70].

Size for each specimen of physically cast steel-reinforced concrete specimens employed for this research work includes 100 mm × 100 mm × 200 mm. Symmetrically placed across the width of each of the blocks was 150 mm out of the 190 mm length of the steel-rebar, which thus have the remaining 40 mm protruding for electrochemical connections. Concrete formulation and casting followed details in reported works [32, 70]. By this, the formulation for each cast concrete block includes 149.7 kg/m^3^ portable water, 300.0 kg/m^3^ Ordinary Portland Cement, 890.6 kg/m^3^ clean sand from Ogun River basin, in Nigeria, and 1106.3 kg/m^3^ granite stones of 19 mm maximum size [71, 72]. By this, the water/cement (w/c) ratio = 0.499 [68]. The casting for the concrete was designed to be in duplicate of specimens, such that the same* R. mangle* L. bark-extract concentration was utilized as admixture in each duplicated concrete slab, as per Corbett [73]. Concentrations of* R. mangle *L. bark-extract admixture in concrete ranged from 0%, in the normal control (Ctrl) specimens, through increments of 0.0833% (one in 1200 parts by weight of* R. mangle* L. bark-extract relative to cement) unto 0.4167%. Apart from these, an additional duplicated specimen of steel-reinforced concrete was cast with 0%* R. mangle* L. concentration for positive control immersion experiments, in distilled water medium [74]. By these, steel-reinforced concrete for this study totalled 14 specimens.

### 2.2. Setup for Corrosion Monitoring Experiment

The 12 normal specimens of steel-reinforced concrete were immersed, partially, along their lengthwise dimension, in 3.5% NaCl-containing plastic bowls, employed for the saline/marine environment simulation [11, 37, 74, 75]. The remaining two specimens of positive control concrete were immersed also in plastic bowls but that contain distilled water, for corroborating that corrosion effects from the NaCl-immersed specimens followed from that test-medium and not from other environmental effects [74]. In the plastic bowls, each of the test-media was ensured to be just below, but without touching, the steel-rebar protruding from the concrete. Steel-rebar corrosion in each of the setup experiments was monitored using the following electrochemical techniques [74, 76, 77]:

- Corrosion-potential (*CP*) versus Model 8-A Cu/CuSO_4_ electrode (CSE), (from Tinker & Rasor®) via connections of a digital multimeter (MASTECH® instrument) having 10 MΩ impedance as per [78].
- Corrosion-current (*CC*) versus CSE via connections of a Model ZM3P zero resistance ammeter (from Corrosion Service®).
- Corrosion-rate (*CR*) measurements via connections of Model MS1500L LPR Data Logger instrument (from Metal Samples®) that employs 3-electrode instrument system for linear polarization resistance applications to the specimen of steel-reinforced concrete. The 3-electrode system is constituted of brass plate for auxiliary, Ag/AgCl SCE for reference (EDT direct-ION®) and the steel-rebar in concrete for working electrodes. By these, direct readout of the* CR* (in mpy unit) is obtained from the setup. The readout in mpy is then converted to mm/y using relationships that were detailed in [35].

These measurements were obtained from the setup of electrochemical monitoring experiments, in five days interval for the initial 40 days and then in seven days interval for the following five weeks. These are such that each of the corrosion test-variables totals 14 data-points of measurements that were taken in the 75 days of experimental period [79].

### 2.3. Analyses of Experimental Data

#### 2.3.1. Applied Statistical Probability Distribution Models and Goodness-of-Fit Studies

As per designation ASTM G16 [80], and the findings from studies [76, 79] that corrosion test-variables could follow either the Normal or the Weibull probability density fitting models, the corrosion test-responses in this study were analysed using the Normal and the Weibull probability distributions. From these, the descriptive statistics of Normal mean,* μ*_*N*_, and standard deviation,* σ*_*N*_, were estimated, for the test-measurement* x*_*i*_ and* n* =14 data-points of test-measurements, using the maximum likelihood estimation (MLE) equations:

(1) μN=1n∑i=1nxi

(2) σN=∑i=1nxi−μN2n−1

The mean,* μ*_*W*_, and standard deviation,* σ*_*W*_, of the Weibull distribution were also estimated from the equations [79, 81, 82]

(3) μW=cΓ1+1k

(4) σW=c2Γ1+2k−Γ1+1k2

where the Weibull shape and scale parameters,* k* and* c,* come from the solution of the simultaneous MLE for the Weibull model given by [82–85]

(5) ∑i=1nln⁡xi−∑i=1nxic^k^ln⁡xic^+nk^−ln⁡c^n=0

(6) c^−∑i=1nxik^n1/k^=0

Also the Kolmogorov-Smirnov goodness-of-fit (K-S GoF) test was utilized for ascertaining scattering of each of the corrosion test-variables to the Normal and the Weibull probability distributions, using the statistics [86–88]

(7) $$\begin{array}{c} D_{n}=D\left(\;x_{1},x_{2},\ldots ,x_{n}\;\right)=\underset{-\infty <x<\infty }{\sup}\left|\;F^{*}\left(\;x\;\right)-F\left(\;x\;\right)\;\right| \end{array}$$

Thus, the electrochemical noise-resistance,* R*_*n*_, was estimated, from the probability distribution model fitting the scatter of* CP* and* CC*, as the ratio of* CP* standard deviation to that of* CC* standard deviation [76, 89]:

(8) $$\begin{array}{c} R_{n}=\frac{\sigma_{CP}}{\sigma_{CC}} \end{array}$$

Also,* θ* (the surface coverage) and* η* (the corrosion-inhibition efficiency) estimations employed the mean model of the probability distribution fitting the scatter of* CR* in the relationships [76, 90, 91]:

(9) θ=CRCtrl sample−CRAdmixed sampleCRCtrl sample

(10) η=CRCtrl sample−CRAdmixed sampleCRCtrl sample×100

#### 2.3.2. Adsorption Isotherm Modelling

The surface coverage,* θ*, was fitted, with* R. mangle *L. bark-extract concentration,* ρ*, to the Langmuir adsorption isotherm [76, 90–92]:

(11) $$\begin{array}{c} \frac{\rho }{\theta }=\frac{1}{K_{ads}}+\rho \end{array}$$

From this fitting in (11), estimation of the* K*_*ads*_, the equilibrium constant for Langmuir absorption-desorption process, was used for modelling* R*_*L*_, the separation factor, and Δ*G*_*ads*_°, the Gibbs free energy for the adsorption model, respectively from

(12) RL=11+KadsCR0

(13) ΔGads∘=log55.5Kads−2.303RT

By the value of the* R*_*L*_, it is possible to indicate that* R. mangle *L. bark-extract adsorption on the studied steel-rebar is irreversible for* R*_*L*_ = 0, or favourable for 0 <* R*_*L*_ < 1, or linear for* R*_*L*_ = 1, or unfavourable for* R*_*L*_ > 1. Similarly, the value of Δ*G*_*ads*_° would be indicative of prevalent physisorption for Δ*G*_*ads*_° around –20 kJ/mol or prevalent chemisorption for Δ*G*_*ads*_° around –40 kJ/mol.

## 3. Results and Discussions

### 3.1. Statistical Probability Distribution Analyses and Compatibility Modelling Results

**Figure 1:**
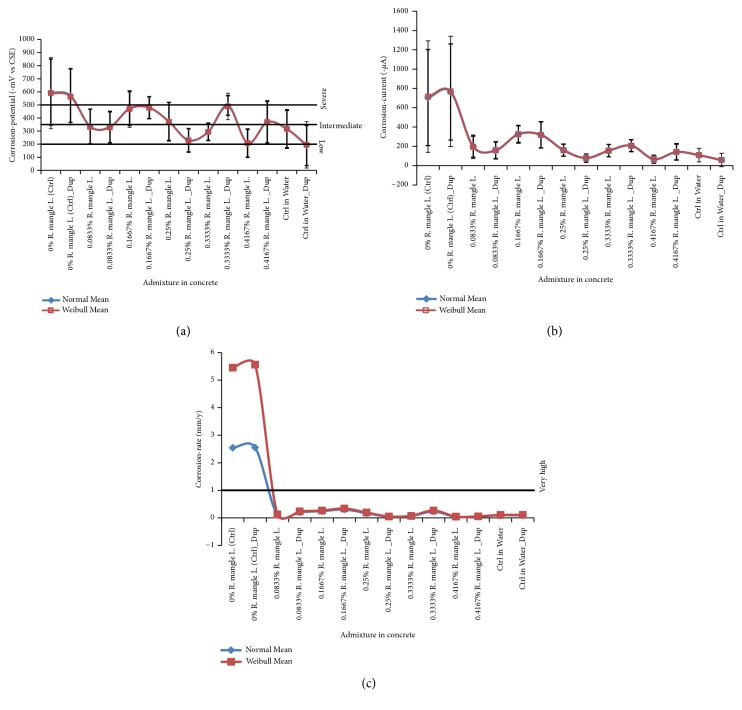
Plots of analysed results from the statistical distribution fitting models of corrosion test-variables: (a) corrosion-potential (*CP*) in mean ± standard deviation ranges with line graphs of corrosion risks criteria according to ASTM C876–15 [78]; (b) corrosion-current (*CC*) in mean ± standard deviation ranges; (c) corrosion-rate (*CR*) in mean values with a line graph of corrosion criteria classification.

The mean ± standard deviation (*μ* ±* σ*) models obtained from the corrosion test-data fittings to the Normal distribution and the Weibull distribution are presented in Figure 1(a) for* CP*, and in Figure 1(b) for* CC*. In Figure 1(c), also, the mean models obtained from fitting* CR *data to these probability distributions are presented. In addition, linear plotting for the corrosion risk criteria according to ASTM C876–15 [78] and for the typical* CR* classification as detailed in the literature [93–95], are respectively shown in Figures 1(a) and 1(c).

Observable from the figure include the fact that despite the different types of corrosion monitoring instruments for the study, values of the corrosion test-variables were higher in the normal control, Ctrl, specimens (in NaCl) than in the specimens admixed with bark-extract of* R. mangle* L. Figure 1(a) indicated that the mean corrosion-potential values for the duplicate of Ctrl specimens were in the “severe” corrosion risk criteria as per ASTM C876–15 [78], a range to which none of the mean corrosion-potential values from the other specimens for the study attained. This is just as the mean corrosion-rate plots in Figure 1(c) from which it is also observable that only the mean corrosion-rate values by the duplicate of Ctrl ranged higher than the “Very high” corrosion classification criteria. In contrasts to these, the corrosion test-variables of* CP*,* CC* and* CR* obtained from the duplicates of positive control in distilled water immersion (Ctrl in Water) were of much more lower values than from the NaCl-immersed (normal) controls. Actually, the mean values from the corrosion test-variables of duplicated specimens having* R. mangle* L. admixture either tend towards or find comparison with mean values of the test-variables for the duplicated specimens of Ctrl in Water. These results imply that the higher valued mean corrosion test-variables in the Ctrl specimens indicate severe corrosion condition in the 3.5% NaCl test-environment for the study, while the low valued corrosion test-responses in the admixed steel-reinforced concrete specimens suggest corrosion-inhibition. The design of severe corrosion condition, as encountered from the results in this study, has been recommended as the preferred practice for laboratory corrosion tests in [96] for reduced timeliness of effects observation, via use of the dominant factor as the rank-ordering factor. According to [96], such practice facilitates comparisons of corrosion resistance in different test-conditions as a function of the factor.

Unlike the plots from the mean Normal distribution model of* CP* and* CC* that patterned well in agreements with Weibull distribution model for these corrosion test-variables, these distribution models of* CR* exhibited discrepancies, especially for the Ctrl specimens. Thus, it is for ascertaining the significance of this form of disparity being observed for which the goodness-of-fit test-statistics is useful, especially, for investigating compatibility of measured data to statistical distribution fittings. Such compatibility testing is prescribed as necessary, by ASTM G16–13 [80], for the avoidance of making erroneous conclusion on the prevalent corrosion condition in the corrosion test-systems, which for this study constitute the specimens of steel-reinforced concrete being tested.

**Figure 2:**
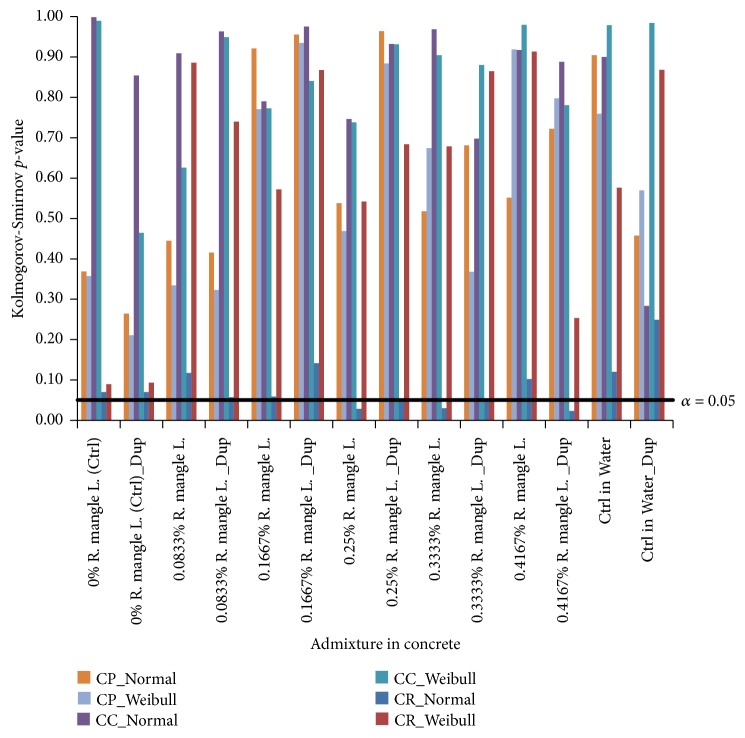
Test-results of compatibility of datasets of corrosion test-variables to the Normal distribution and to the Weibull distribution by the Kolmogorov-Smirnov goodness-of-fit statistics.

Thus, Figure 2 presents the plots of findings from the Kolmogorov-Smirnov goodness-of-fit analyses applied to the fitting of the datasets of corrosion test-variables obtained from each steel-reinforced concrete to the Normal distribution and to the Weibull distribution. Also, the linear plot of *α* = 0.05 significant level criteria has been included in the Figure 2, for directly identifying dataset that scattered like each of the distribution model, or otherwise.

This figure, therefore, showed that while the Kolmogorov-Smirnov goodness-of-fit test-criteria indicate that the* CP* and* CC* datasets for all the studied steel-reinforced concrete scattered like both the Normal and the Weibull models, four* CR* datasets did not follow the Normal distribution. Included among these are the* CR* datasets from the 0.25%* R. mangle *L., the 0.3333%* R. mangle *L. the 0.3333%* R. mangle *L. Dup and the 0.4167%* R. mangle *L. bark-extract admixed steel-reinforced concrete specimens. However, the* CR *datasets from these and the remaining specimens of studied steel-reinforced concrete followed the Weibull distribution, just as the* CC* and* CP* datasets from the experimental specimens. These compatibilities of all the corrosion test-variable datasets to the Weibull probability distribution support the Weibull distribution usage for describing and detailing the corrosion condition of the test-systems in this study.

### 3.2. Results from Corrosion Test-Variable Modelling

**Figure 3:**
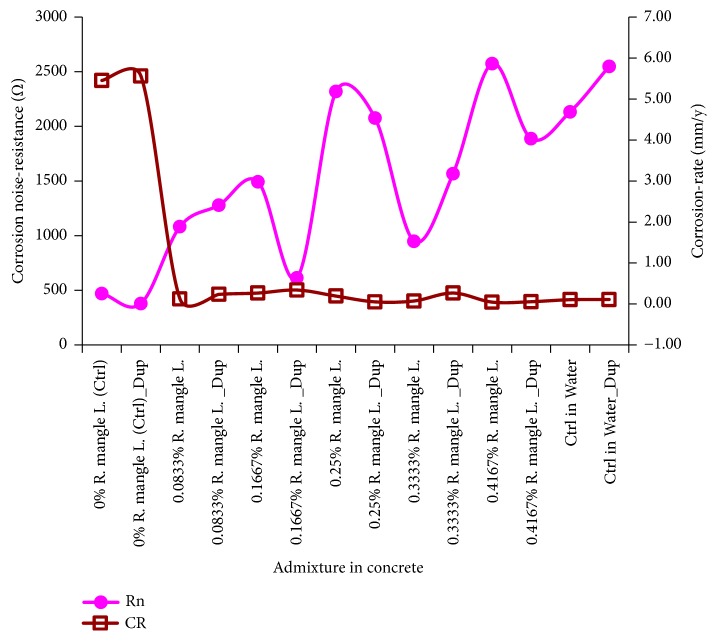
Electrochemical noise-resistance superimposed on corrosion-rate from specimens of steel-reinforced concrete.

In Figure 3 are shown plot of electrochemical noise-resistance, the ratio of the standard deviation of corrosion-potential to that of the corrosion-current, via the Weibull models of these test-variables, superimposed on the mean model of corrosion-rate also by Weibull distribution.

Observable from this figure include the fact that apart for some fluctuations, the electrochemical noise-resistance exhibited an increasing trend as the* R. mangle *L. bark-extract admixture concentration increases, at which corrosion-rate tends to decrease. The lowest electrochemical noise-resistance models in the study were obtained from the NaCl-immersed Ctrl (normal control) specimens, which are the specimens from which the highest values of corrosion-rate models were also obtained. From these Ctrl specimens, the trend of the corrosion-noise resistance increases even as it fluctuates through the increasing* R. mangle *L. bark-extract concentrations. Also, just as the corrosion-rate from the duplicates with 0.4167%* R. mangle* L. bark-extract admixture, the electrochemical noise-resistance values modelled from these specimens compare well with the values obtained from the duplicate of Ctrl in Water (positive control) specimens. These suggest corroborations of low corrosion condition in the specimens of steel-reinforced concrete with the 0.4167%* R. mangle* L. bark-extract, just as was observed from the Ctrl in Water specimens, by the different types of instruments employed for the study. These trends conform to findings in the literature [74, 79, 97] in which low corrosion-rate is attended by high electrochemical noise-resistance and vice versa, thus suggesting inverse tracking or correlation relationship between these models of corrosion variables.

### 3.3. Correlation Modelling of Corrosion Test-Results

Investigation of mathematical-correlation between the corrosion-rate,* CR*, as independent variable,* R. mangle* L. bark-extract concentration,* ρ*, and the electrochemical noise-resistance as the dependent variables gives the relationship that could be express in compact form as

(14) $$\begin{array}{c} CR=0.6094\left[\;\rho +\overset{5}{\underset{\xi =0}{\sum }}{\left(\;-1\;\right)}^{\xi +1}\cdot a_{\xi }\cdot 10^{3\xi }\cdot {\left(\;1/R_{n}\;\right)}^{\xi }\;\right] \end{array}$$

where the values of the coefficients *a*_*ξ*_, {*ξ* = 0,1, 2,…, 5}, are detailed in Table 1.

For the obtained model of mathematical-correlation relationship in (14)* R* (the correlation coefficient) = 99.92% and* NSE* (the Nash-Sutcliffe efficiency) = 99.96%. These modelling parameters interpret to excellent model efficiency, in accordance with classification for this in Coffey et al. [98]. From the analysis of variance for the modelled correlation, in Table 2, the ANOVA* p*-value = 1.2125×10^–7^. This being less than 0.05 by the* p*-value supports the existence of statistically significant relationship between the dependent variable of corrosion-rate (*CR*) and the studied independent variables.

It is worth noting from these correlation test-results that* Phyllanthus muellerianus* leaf-extract, which has been used for inhibiting chloride-induced corrosion of concrete steel-rebar, also fitted excellently to the compact form correlation equation (14) [74]. This similarity in corrosion behaviour model-relationship could be due to these two admixtures being extracts from natural-plants, though the extract from* Phyllanthus muellerianus *was from its leaf while the extract for this study was from the* R. mangle *L. bark. However, the constants and the parameters of correlation from that work on* Phyllanthus muellerianus* leaf-extract are different in values, which are mostly lower than the values obtained from the present case. The exception to these lower-valued parameters, from the* Phyllanthus muellerianus* leaf-extract and* R. mangle *bark-extract corrosion behaviour model comparisons, is the ANOVA* p*-value from this study that is (advantageously) lower than the* p-value* from that work. This lower* p-value* obtained presently indicates the fitting of (14) by the experimental data in this study exhibited higher level of statistical significance than the reported fitting of this same compact form equation by* Phyllanthus muellerianus* leaf-extract.

### 3.4. Corrosion-Inhibition Modelling from the Experimental Data and Mathematical-Correlation

**Figure 4:**
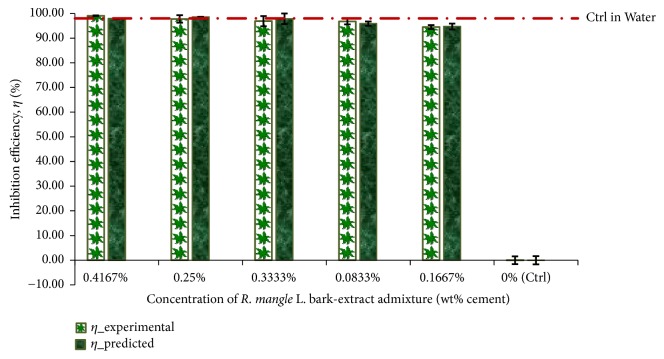
Plots of corrosion-inhibition efficiency for the specimens of steel-reinforced concrete studied.

Applying the experimental mean model (by Weibull) of corrosion-rate and the corrosion-rate predicted from the correlation model of (14) to corrosion-inhibition efficiency modelling equation (10) gives the ranking of inhibition performance presented in Figure 4. The plotted values in the figure have been averaged over each duplicate of specimens having the same* R. mangle *L. bark-extract admixture concentration. Also shown in the figure include the plot of the linear graph of the reduction in corrosion effect from the positive control specimens averaged over their duplicates, which was idealised as inhibition efficiency with reference to the corrosion effect from the normal control (Ctrl). This was done for showing the two modes of corrosion effect comparisons employed for this study, i.e., the comparison relative to the normal control as well as the comparison relative to the positive control specimens.

Therefore, by the comparison relative to the normal controls, all the admixture concentrations of* R. mangle* L. bark-extract for the study exhibited excellent corrosion-inhibition performance,* η* > 90%, both from the conducted experiment and the predictions from the correlation model. From another consideration, the higher* R. mangle *L. bark-extract concentrations, the 0.1467%, 0.3333%, and 0.25%, exhibited ranges of corrosion-inhibition effects that either surpassed or compared well with the corrosion reduction effects from the positive (water-immersed) control specimens. The remaining two concentrations of bark-extract from* R. mangle *L. for the study, i.e., the 0.1667% and the 0.0833%, exhibited corrosion-inhibition effects that just fall short of the reduced corrosion effects exhibited by the positive control specimens. These further supports that the 3.5% NaCl medium employed in the study constitute a corrosive environment and that the corrosion-inhibition performance observed from the admixed steel-reinforced concrete followed from the bark-extract admixture from* R. mangle* L. in the concrete.

From the ranking of corrosion-inhibition performance plotted in Figure 4, therefore, the bark-extract admixture from* R. mangle *L. exhibited the highest corrosion-inhibition performance,* η* = 99.08±0.11% (experimental) or 97.89±0.24% (correlation), in the study. In comparison, the corrosion reduction effect observed from the Ctrl in Water idealised to* η* = 98.03±0.05% relative to the NaCl-immersed normal control specimens. This showed that the experimental performance from the* R. mangle *L. bark-extract surpassed (while the correlation prediction exhibited ranges that find good comparisons with) the reduction effects in corrosion that was exhibited by the Ctrl in Water concrete.

These anticorrosion performance effects by* R. mangle* L. on concrete steel-rebar bare suggestions that the biochemical compounds characterised from* R. mangle *L. bark-extract in [56] also exhibited pore-blocking and film-forming properties in addition to corrosion-inhibition [95]. The biochemical compounds detailed in [56] include amines, such as CH_5_NO_3_S (aminomethanesulfonic acid), CH_8_Cl_2_N_2_ (methylenediamine dihydrochloride), C_3_H_7_NO_2_ (alanine) and C_12_H_12_NO_2_P ((aminooxy)(diphenyl)phosphine oxide, or O-(Diphenylphosphinyl) hydroxylamine). These groups are known to exhibit pore-blocking characteristics, in addition to corrosion inhibiting properties [93]. Also, the biochemical constituent characterised from* R. mangle *L. include C_16_H_26_O_3_, i.e., methyl (2E,6E)-(10R)-10,11-epoxy-3,7,11-trimethyl-2,6-dodecadienoate. This compound belongs to the ester group of organic chemistry. According to the literature [93, 99, 100], ester compounds hydrolyze in the alkaline mix of concrete casting into hydrophobic salts that resist water penetration into concrete. Consequently, such hydrophobicity also precludes penetration of the aqueous medium of corrosive agent into the concrete pore environment, in which the steel-rebar is embedded.

### 3.5. Adsorption Isotherm Fitting of Experimental and Correlation Performance Modelling 

**Figure 5:**
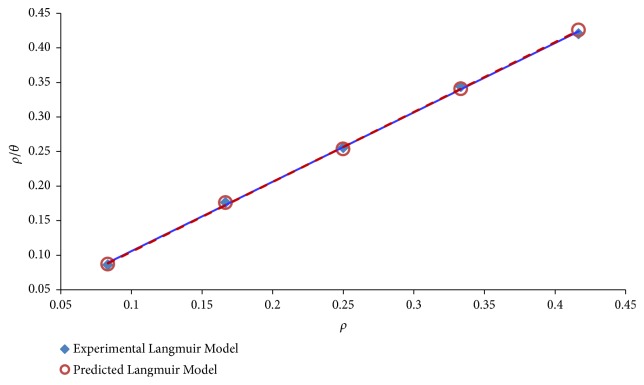
Langmuir adsorption isotherm fitting of experimental and correlation data.

The Langmuir adsorption isotherm fitting of experimental and correlation performances, as per (9) and (11), gives the plots presented in Figure 5. This shows that the fitting assumed straight line adsorption isotherm model for each of the experimental and correlation prediction. The fitting parameter ensuing from this modelling are presented in Table 3, from which the correlation coefficient* R* = 99.97% (experimental) or 99.98% (correlation). Both of these interpret to excellent model fitting in accordance with the model efficiency classification of [98].

From Table 3, the separation factor,* R*_*L*_ = 1.0221 × 10^–3^ (experimental) or 7.1166 × 10^–4^ (correlation). That these are in the range 0 <* R*_*L*_ < 1 suggests favourable adsorption model by the* R. mangle* L. bark-extract on the steel-rebar, in accordance with the separation factor classification in [92]. Also, value of the Gibbs free energy of adsorption, Δ*G*_*ads*_ was negative, and with the value being around 20 kJ/mol, for each of the experimental and correlation models. These respectively indicate spontaneity of the adsorption process and prevalent physisorption as the mechanism of* R. mangle *L. bark-extract corrosion-protection on the concrete steel-rebar.

## 4. Conclusions

In this paper,* R. mangle* L. bark-extract behaviour on the inhibition of steel-rebar corrosion in concrete specimens immersed in 3.5% NaCl, for simulating a saline/marine environment, has been studied. In the work, statistical analyses of the corrosion test-results, by the Weibull distribution model that the corrosion test-data preferentially followed, showed excellent mathematical-correlation between corrosion-rate, the* R. mangle* L. bark-extract admixture concentration and corrosion-noise resistance. For this excellent mathematical-correlation of corrosion test-variables from different instrumentation, correlation coefficient* R* = 99.92%, Nash-Sutcliffe Efficiency = 99.96%, and ANOVA* p*-value = 1.2125×10^–7^. By these, the 0.4667%* R. mangle* L. bark-extract admixture was the optimally efficient admixture concentration from the steel-rebar corrosion-inhibition experiments in the study with the inhibition efficiencies,* η* = 99.08±0.11% (experimental) or* η* = 97.89±0.24% (correlation). The inhibition efficiency by this admixture concentration even experimentally outperformed the inhibition efficiency model from the positive control specimens immersed in distilled water environment. Fitting of the experimental data and predicted results from the mathematical-correlation to Langmuir adsorption isotherm indicates spontaneous/favourable adsorption and prevalence of physisorption as the corrosion-protection mechanism by this natural plant-extract on steel-rebar metal. Based on these findings, it is established in this study that the bark-extract from* R. mangle* L. natural plant will be suitable as a green/environmentally friendly admixture for steel-rebar corrosion-protection in concrete designed for the saline/marine service-environment.

## Figures and Tables

**Table 1:** Values of coefficients *a*_*ξ*_ for (14).

*ξ*	*a*_*ξ*_
0	6.7566
1	43.5567
2	102.8128
3	106.8378
4	48.7760
5	7.8202

**Table 2:** ANOVA for the modelled correlation in (14).

Source of variation	DoF	SS	MS	*F*	*p*-value
Treatment	6	47.6323	7.9387	1110.0372	*1.2125×10* ^*–7*^
Residual	5	0.0358	0.0072		
Total	11	47.6680			

**Table 3:** Modelled Langmuir adsorption isotherm fitting parameters.

Isotherm parameter	Experimental model	Predicted model
*K*_*ads*_	177.4666	255.0057
*R*, correlation coefficient, (%)	99.97	99.98
*R*_*L*_, separation factor	1.0221 × 10^–3^	7.1166 × 10^–4^
Δ*G*_*ads*_ Gibbs free energy (kJ/mol)	–27.6209	–23.8387

## Data Availability

The data used to support the findings of this study are included within the article.
